# Hypotension and pulmonary atelectasis during general anaesthesia: a randomised crossover laboratory study

**DOI:** 10.1016/j.bja.2026.05.008

**Published:** 2026-06-24

**Authors:** Erland Östberg, Cecilia Wassberg, Mark Lubberink, Jonathan Sigfridsson, Miklós Lipcsey, Gaetano Perchiazzi, Philippe Wagner, Lennart Edmark

**Affiliations:** 1 Department of Anaesthesia and Intensive Care, Västmanland Hospital Västerås, Sweden; 2 Centre for Clinical Research Västmanland, Department of Surgical Sciences, Uppsala University, Sweden; 3 Molecular Imaging and Medical Physics, Department of Surgical Sciences, Uppsala University, Sweden; 4 Uppsala Centre for Molecular Imaging and Theranostics, Department of Surgical Sciences, Uppsala University, Sweden; 5 Anaesthesiology and Intensive Care, Department of Surgical Sciences, Uppsala University, Sweden; 6 Hedenstierna Laboratory, Department of Surgical Sciences, Uppsala University, Sweden; 7 Department of Anaesthesia, Operation and Intensive Care, Uppsala University Hospital, Uppsala, Sweden

**Keywords:** general anaesthesia, hypotension, mechanical ventilation, mixed venous oxygen saturation, pulmonary atelectasis, pulmonary blood flow

## Abstract

**Background:**

Atelectasis is common during general anaesthesia and contributes to postoperative pulmonary complications. Despite preventive strategies targeting established underlying mechanisms, extensive atelectasis still often occurs, suggesting that additional factors may be involved. We tested the hypothesis that hypotension contributes to atelectasis formation during anaesthesia.

**Methods:**

We conducted a randomised, evaluator-blinded crossover study in 12 anaesthetised, mechanically ventilated pigs under conditions simulating anaesthesia induction, in which preoxygenation was followed by either normotension or profound hypotension induced with nitroprusside. The primary outcome was total atelectasis volume, measured by computed tomography. Pulmonary blood flow distribution was assessed using [^15^O]-water positron emission tomography.

**Results:**

Atelectasis volume was greater during induced hypotension (median MAP 46 mmHg [interquartile range: 43–49]), compared with normotension (median MAP 85 mmHg [81–93]) in 11 of 12 pigs (median difference 20 ml [95% CI 9–38]; *P*<0.001). During hypotension, pulmonary blood flow was redistributed towards the dorsal lung regions, with hypotension resulting in a ratio of dorsal to total pulmonary blood flow of 182% (170–186), compared with 129% (104–150) with normotension. Mixed venous oxygen saturation was lower during hypotension (43% [27–53]) compared with normotension (48% [44–56]).

**Conclusions:**

In this experimental model of anaesthesia induction, profound hypotension increased atelectasis compared with normotension. The findings are consistent with redistribution of pulmonary blood flow with low oxygen saturation towards dorsal lung regions that are prone to develop absorption atelectasis. Hypotension during induction of general anaesthesia might therefore be a hitherto unrecognised contributor to pulmonary atelectasis.

**Trial registration:**

www.animalstudyregistry.org (https://doi.org/10.17590/asr.0000369).

**Editor’s key points**

• The aetiology of atelectasis and its ensuing postoperative pulmonary complications remains incompletely understood.
• The authors examined whether hypotension contributes to atelectasis formation.
• In an experimental porcine model, profound hypotension during induction of anaesthesia increased atelectasis formation, despite identical ventilatory conditions.
• Hypotension redistributed pulmonary blood flow towards dependent dorsal lung regions that are vulnerable to absorption atelectasis.
• These experimental findings identify hypotension as a potential modifiable contributor to perioperative atelectasis, warranting further clinical investigation.

Pulmonary atelectasis occurs in most adult patients undergoing general anaesthesia.^1^ The collapse of lung alveoli impairs oxygenation through shunt formation and reduces pulmonary compliance by decreasing the volume of aerated lung. Atelectasis often persists after surgery and contributes to hypoxaemia and possibly pneumonia.^2^ Accordingly, preventing atelectasis is a key target of intraoperative ventilation strategies aimed at reducing postoperative pulmonary complications, an important cause of morbidity and mortality.^3^^,^^4^

Several factors contribute to atelectasis.^5^ Research has largely focused on two pulmonary determinants associated with induction of general anaesthesia: loss of respiratory muscle tone, leading to reduced end-expiratory lung volume and airway closure, and elevated alveolar oxygen concentration resulting from preoxygenation. Accordingly, various ventilation strategies have been evaluated to counteract these effects, including the use of PEEP and lung recruitment manoeuvres to restore lung volume. Modulating the oxygen concentration during preoxygenation has also been examined, although reducing it inevitably shortens the safe apnoea time.^6^

Despite these strategies, some patients still develop extensive atelectasis, suggesting that additional mechanisms may be involved. The potential contribution of the circulatory system has received little direct investigation, although it has been considered in theoretical modelling.^7^ An early experimental study reported that hypotension was associated with increased venous admixture and reduced lung compliance,^8^ findings consistent with atelectasis as a possible underlying mechanism. Moreover, two clinical studies reported greater-than-anticipated atelectasis in patients with profound postinduction hypotension, suggesting that systemic haemodynamics may influence atelectasis development.^9^^,^^10^ Systemic hypotension may alter pulmonary blood flow distribution and mixed venous oxygen content, both of which could contribute to absorption atelectasis.

We therefore designed an experimental study to investigate whether hypotension contributes to atelectasis formation during anaesthesia.

## Methods

### Study design

This was a randomised, evaluator-blinded crossover laboratory study in 12 anaesthetised and mechanically ventilated pigs. The study was conducted at the Hedenstierna Laboratory, Uppsala, Sweden, between Nov 2024 and April 2025, and in accordance with the ‘Guide for the Care and Use of Laboratory Animals’ (EU Directive 2010/63). Pigs were selected as a suitable species because their respiratory and cardiovascular systems closely resemble those of humans, and studies have indicated that they develop atelectasis by the same mechanisms as humans.^11^^,^^12^ The crossover design was chosen to maximise statistical power while reducing the number of animals required, in accordance with the principle of reduction in animal research (3R). All efforts were made to minimise animal suffering. Adequacy of anaesthesia was ensured by monitoring vital signs and confirming absence of movement or response to noxious stimulation throughout the experiment. At the conclusion of the experiments, all animals were euthanised with an i.v. injection of potassium chloride while still under deep anaesthesia.

The study was approved by The Regional Animal Ethics Committee in Uppsala (approval number 5.8.18-09415/2023). The reporting complied with relevant aspects of the Animal Research Reporting In Vivo Experiments (ARRIVE) guidelines.^13^ The study protocol and statistical analysis plan were pre-registered at www.animalstudyregistry.org (https://doi.org/10.17590/asr.0000369).

### Randomisation

All animals underwent both study conditions and were randomly assigned (1:1) to study sequences using block randomisation (block size four) to ensure balanced allocation while limiting predictability. An independent research assistant generated the computer-based randomisation sequence and prepared sealed allocation envelopes in advance. Allocation was revealed immediately before the first simulated anaesthesia induction.

### Procedures

On arrival at the laboratory, the pigs were sedated with an i.m. injection of xylazine (2.2 mg kg^−1^) and tiletamine-zolazepam (6 mg kg^−1^) to tolerate venous access. After an i.v. dose of fentanyl, the animals were positioned supine, and a tracheostomy was performed with insertion of a shortened endotracheal tube during spontaneous breathing on room air or, if necessary, supplemental oxygen to achieve an SpO_2_ of 90–94%. Intravenous anaesthesia was induced and maintained with ketamine 32 mg kg^−1^ h^−1^, midazolam 0.12 mg kg^−1^ h^−1^, and fentanyl 4 μg kg^−1^ h^−1^. Neuromuscular block was maintained with rocuronium 2.5 mg kg^−1^ h^−1^. A bolus dose of 600 ml Ringer´s lactate was given at induction, followed by fluid maintenance with 2.5% glucose 8 ml kg^−1^ h^−1^.

Mechanical ventilation was performed using a Servo-I ventilator (Maquet Critical Care, Sweden) in volume-controlled mode with a tidal volume of 8 ml kg^−1^, PEEP of 5 cm H_2_O, an inspiratory-to-expiratory ratio (I:E) of 1:2, and a respiratory rate of 25–30 bpm, adjusted to maintain an end-tidal carbon dioxide (ETCO_2_) concentration of 5–6%. Plateau pressures were recorded continuously. The maintenance fraction of inspired oxygen (FiO_2_) was set at 0.3 throughout the experimental protocol.

Arterial and central venous pressures were measured via a cervical artery and a central venous catheter, respectively. MAP was maintained at 70–90 mm Hg using low-dose norepinephrine infusion as required. A pulmonary artery catheter was used for continuous pulmonary artery pressure monitoring and CO measurement by the thermodilution technique. Arterial and mixed venous blood samples were obtained and analysed repeatedly using the Radiometer ABL 800 Flex (Radiometer Medical, Denmark). Venous admixture, defined as the sum of true shunt and blood originating from poorly ventilated lung regions, was calculated using the standard shunt equation.^14^

After animal preparation, an initial lung recruitment was performed in pressure-controlled mode with FiO_2_ 0.3, a driving pressure of 25 cm H_2_O, a PEEP of 15 cm H_2_O, and I:E 1:1. The respiratory rate was set to 6 bpm for 1 to 2 breaths, followed by a 30 s inspiratory hold. A 45-min stabilisation period preceded the start of the experiments.

### Experimental protocol

At the positron emission tomography (PET) centre, the study animals were positioned in the PET–computed tomography (PET-CT) scanner (Discovery MI-5, GE Healthcare, Chicago, IL, USA), remaining supine. A second lung recruitment was performed before PET-CT 1 (baseline). The study protocol was designed to simulate the respiratory changes accompanying induction of anaesthesia: preoxygenation, loss of lung volume, and a brief period of apnoea, followed by either normotension (*control* condition) or hypotension (*intervention* condition).

In the intervention condition, hypotension to a target MAP of 35 mm Hg was achieved with an i.v. infusion of nitroprusside 1.1 mg ml^−1^, in a syringe also containing sodium thiosulphate 11 mg ml^−1^ to eliminate the risk of cyanide production.^15^ The onset of induced hypotension was timed to mirror clinical practice, beginning after a standard 3 min preoxygenation with FiO_2_ 1.0 and at the start of i.v. anaesthesia induction. The nitroprusside infusion was initiated at 60 ml h^−1^ and adjusted to reach the target MAP within ∼ 5 min. Once this target was reached, the infusion rate was modified only if MAP remained below 35 mm Hg; otherwise, it was kept at its set value for the duration of the subsequent PET scan. The infusion was then stopped, resulting in a total hypotension duration of 10–12 min, roughly simulating the duration of postinduction hypotension in clinical practice while avoiding haemodynamic fluctuations during PET acquisition.

**Fig 1:**
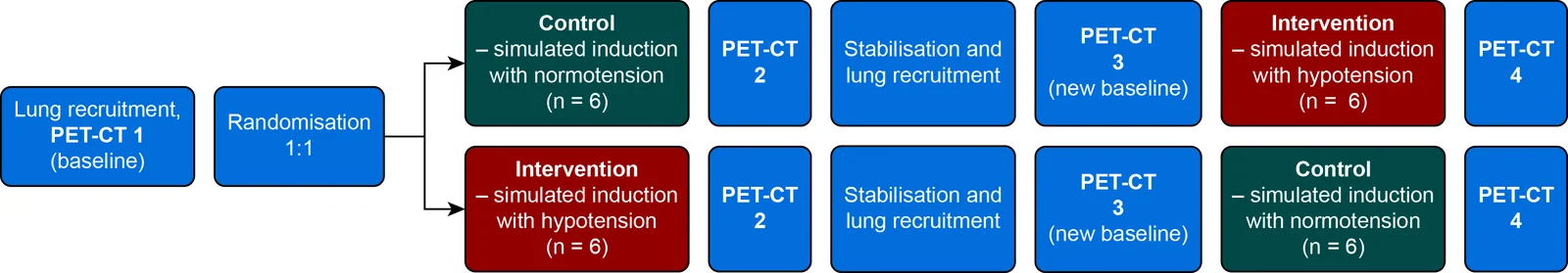
Schematic overview of the study timeline and randomised crossover study design. All 12 animals underwent both study conditions. PET-CT, Positron emission tomography – computed tomography.

**Fig 2:**
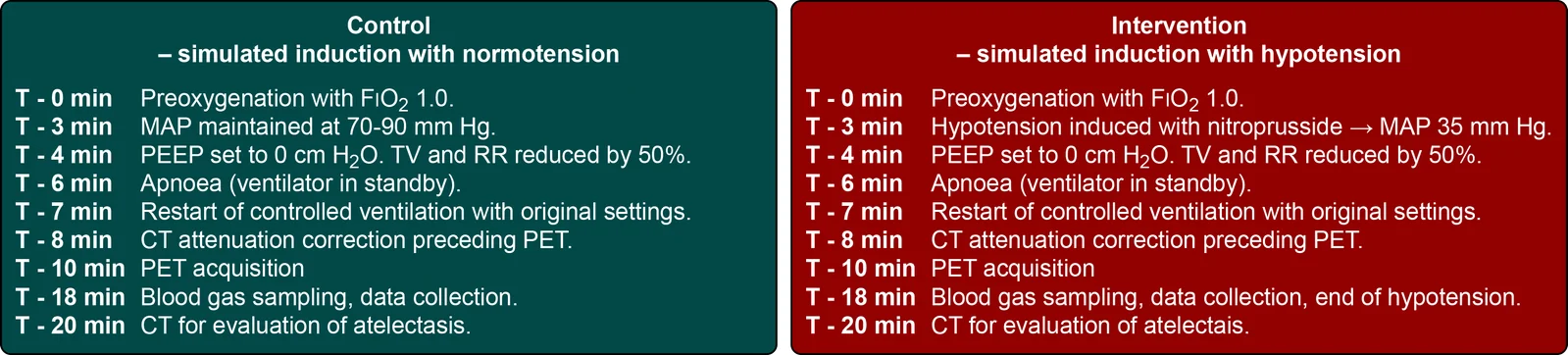
Time sequence of simulated anaesthesia induction protocols under *control* (normotension) and *intervention* (hypotension). FiO_2_, fraction of inspired oxygen; TV, tidal volume; RR, respiratory rate; PET, positron emission tomography.

To minimise carryover effects between the two study conditions, a third recruitment manoeuvre was performed between sessions, followed by a CT scan to establish a new baseline before each control or intervention session. The study timeline is shown in Figure 1, and the simulated anaesthesia induction sequences are detailed in Figure 2.

### Computed tomography

CT scanning was performed with maintained PEEP and with the tracheal tube clamped at end-expiration. Four thoracic CT series were acquired for each subject. Obtained images were reconstructed to 2.5 mm to optimise image quality and ensure consistency in subsequent analyses.

From each CT series, 10 consecutive, evenly spaced transaxial slices (1 cm apart) were selected from a region extending from 4 cm above to 5 cm below the diaphragmatic dome, ensuring consistent coverage of the lung bases. This region was chosen to maximise measurement sensitivity, as atelectasis in all study animals occurred almost exclusively within this basal lung zone.

Atelectatic regions were delineated using a semi-quantitative threshold-based approach on a GE Advanced Workstation (AW Server 3.2 GE Healthcare). Lung tissue with Hounsfield unit values between −100 and +100 was classified as atelectasis. Atelectatic regions were manually outlined, and vascular structures larger than 5 mm in diameter were identified and manually excluded from the regions of interest. Atelectasis volume was first quantified for each individual slice. Total atelectasis volume within the analysed lung region was then calculated by extrapolation from the 10 slices, according to the method described by Reske and colleagues.^16^ All CT series were evaluated by a single radiologist who was blinded to study condition and outcomes.

### Positron emission tomography

After a CT scan for attenuation correction, 400 MBq kg^−1 15^O-water was administered i.v. as an automated bolus (10 ml at 0.8 ml s^−1^ followed by 30 ml saline at 2 ml s^−1^), initiated simultaneously with a 4-min PET acquisition. Images were reconstructed using time-of-flight ordered-subset expectation maximisation (3 iterations, 16 subsets) with a 5 mm Gaussian post-filter, yielding 20 dynamic frames on a 300 mm field-of-view with 128×128 matrix. All standard corrections required for quantitative imaging were applied.

The pulmonary circulation input function was derived by placing a 1-cm^3^ volume of interest (VOI) within the right ventricle on the image frame in which it was best visualised and propagating this VOI across all dynamic frames. Pulmonary blood flow images were generated using a basis function implementation of a single-tissue compartment model.^17^^,^^18^ A lung mask was defined to include all voxels with Hounsfield units between −900 and −100. Mean total pulmonary blood flow (ml cm^−3^ min^−1^) was calculated across the entire lung mask. Regional pulmonary blood flow was derived by dividing the lung mask into three equally thick ventral, intermediate, and dorsal sections. Pulmonary blood flow distribution was expressed as the ratio of mean regional (ventral, intermediate, and dorsal) to mean total blood flow (%). Because perfusion is regionally heterogeneous, ratios may exceed 100%, reflecting regional flow values greater than the mean total pulmonary blood flow.

### Primary outcome

The primary outcome was the change in total atelectasis volume (ml) after each simulated anaesthesia induction (with and without hypotension), calculated relative to the corresponding baseline. Accordingly, baseline atelectasis volume was subtracted from the volume measured after the control and intervention conditions.

### Secondary outcomes

Secondary outcomes were pulmonary blood flow distribution, mixed venous oxygen saturation (SvO_2_), and venous admixture under the two study conditions. Data on haemodynamics, ventilation, and gas exchange were recorded immediately after each PET acquisition, corresponding to approximately 18 min after simulation onset.

### Statistical analysis

In the crossover design, in which each animal underwent both control and intervention conditions, Monte Carlo simulations indicated that 12 animals would provide sufficient power to detect a large difference in atelectasis volume. The power analysis assumed a standardised effect size of 1 (Cohen’s d), used to represent the expected magnitude of the effect in the simulations, and was informed by physiological considerations and prior observations of hypotension-associated atelectasis.^9^^,^^10^ The analysis used the exact Wilcoxon signed-rank test, yielding an estimated power of 0.83. Data for each study condition are presented as median (IQR). Paired differences are reported as Hodges–Lehmann estimates with 95% confidence intervals. Primary and secondary outcomes were analysed using the exact Wilcoxon signed-rank test.

The associations between atelectasis volume and SvO_2_, and venous admixture, were estimated using Spearman’s rank correlation across both study conditions. To account for the crossover design with repeated measurements per animal, clustered bootstraps were used to obtain 95% CIs. Sensitivity analyses for potential carryover and period effects were also performed using the exact Wilcoxon Rank-Sum Test, acknowledging limited power in this small study sample.

All tests were two-sided and a *P*-value <0.05 was considered statistically significant. All analyses were performed in R, version 4.3.3 (R Core Team, R Foundation for Statistical Computing, Austria).

## Results

### Study flow

Twelve juvenile pigs (Hampshire–Yorkshire–Norwegian Landrace; seven males and five females; 28–34 kg) were included. All completed the study without protocol-related complications. The first baseline PET-CT scan was acquired in one animal (randomised to start with the control condition) without the preceding second lung recruitment manoeuvre, and one cardiac output measurement failed during the intervention condition in one animal. No other data were missing.

### Primary outcome: atelectasis volume

**Fig 3:**
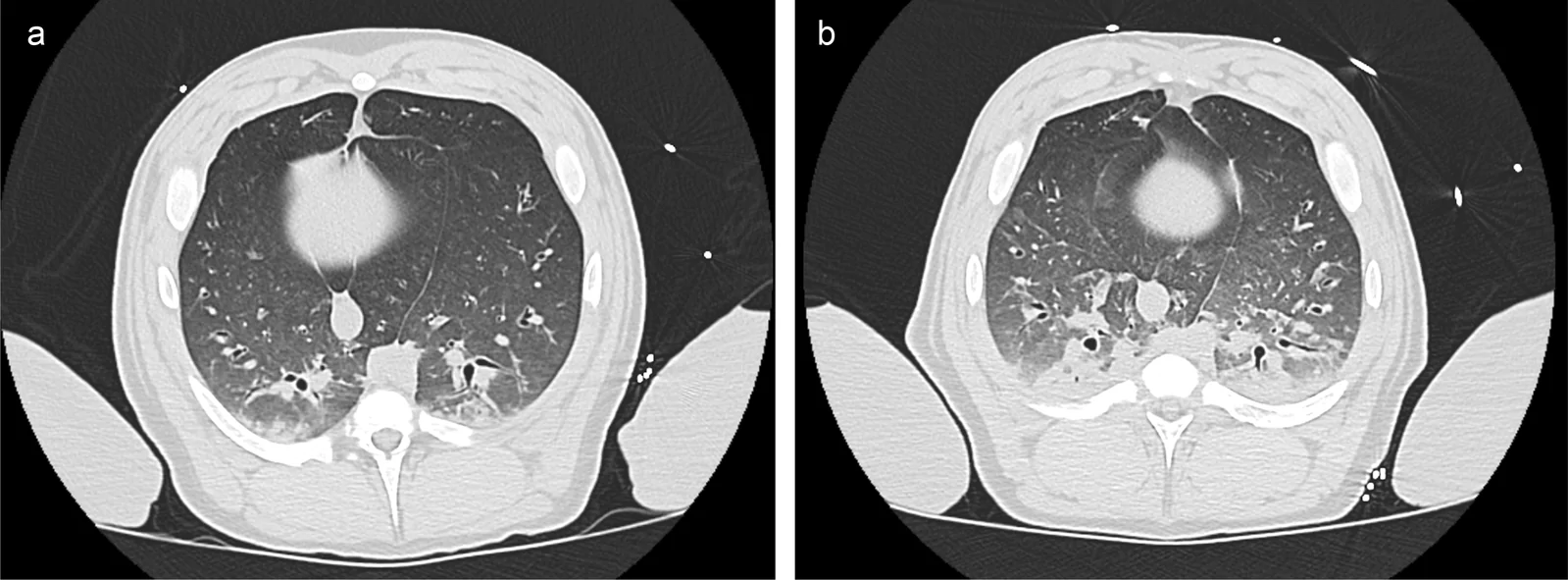
Representative CT scans under *control* (normotension; a) and *intervention* (hypotension; b). Images were selected from animals with total atelectasis volumes close to the respective group medians. These examples show a single slice; the primary outcome was change in total atelectasis volume after each simulated anaesthesia induction relative to baseline.

Total atelectasis volume was greater during hypotension than normotension in 11 of 12 pigs and unchanged in one pig. The median change from baseline was 76 ml (IQR 56–91) *vs* 51 ml (29–70), median difference (Hodges–Lehmann estimate) 20 ml (95% CI 9–38; *P*<0.001). The baseline atelectasis volume subtracted from each study condition was 2.5 ml (IQR 1–10) before the control condition and 3.0 ml (2–11) before the intervention condition, median difference 0.5 ml (95% CI −8.5 to 5.5; *P*=0.760). Representative CT scans from the two study conditions are shown in Figure 3.

### Secondary outcomes

**Fig 4:**
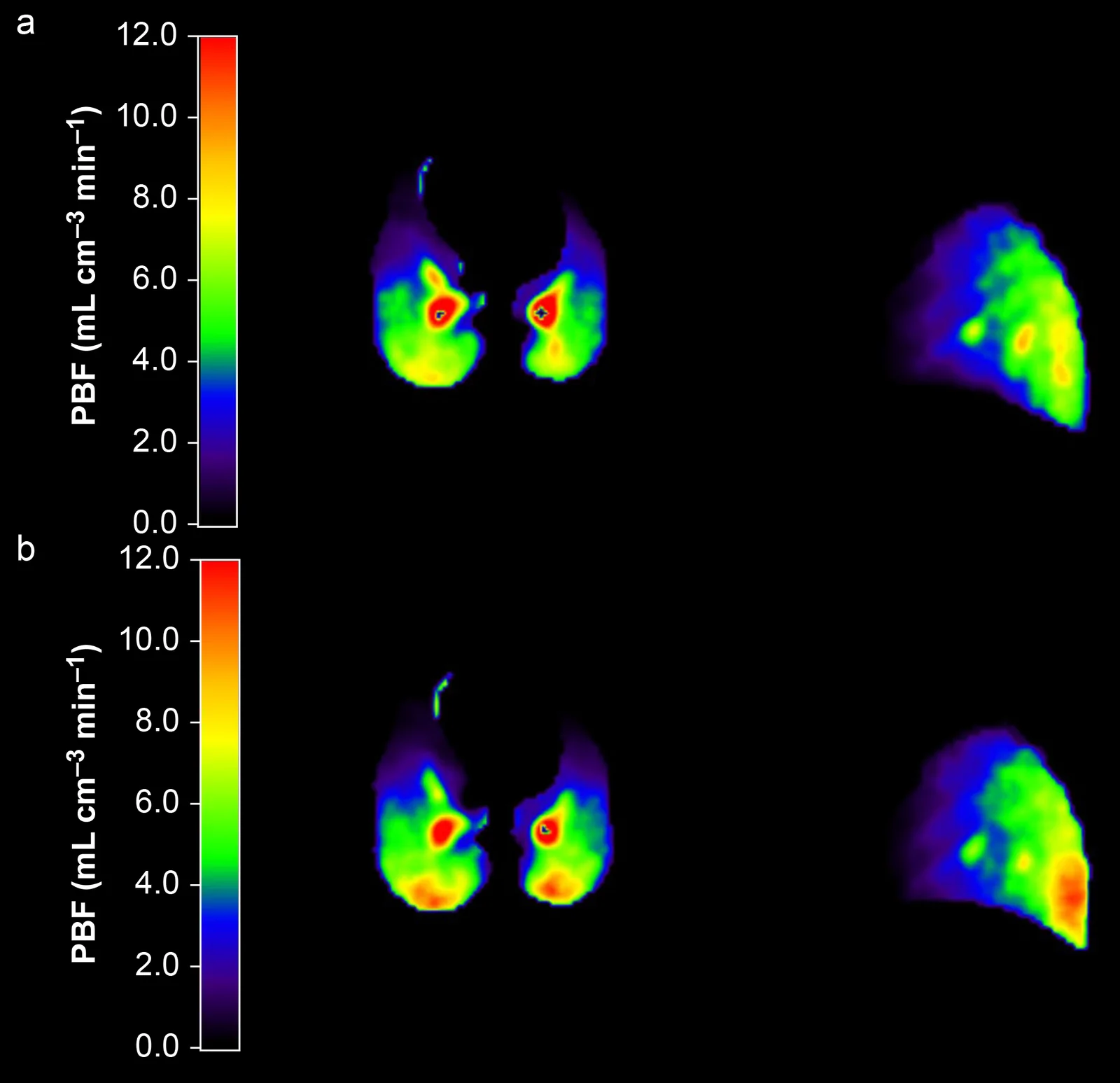
Representative positron emission tomography scans illustrating pulmonary blood flow during *control* (normotension; a) and *intervention* (hypotension; b). In axial (left) and sagittal (right) views, greater perfusion (red) is observed in dorsal lung regions during hypotension. PBF, pulmonary blood flow.

At baseline, the distribution of pulmonary blood flow, expressed as the ratio of mean ventral, intermediate, and dorsal blood flow to mean total pulmonary blood flow, was median 24% (IQR 23–28), 97% (92–109), and 128% (95–150), respectively. These values were comparable with those observed during normotension (27% [22–31], 95% [91–106], and 129% [104–150]). By contrast, during hypotension, all pigs demonstrated a redistribution of pulmonary blood flow away from the ventral and intermediate regions towards the dorsal region, with corresponding values of 20% (18–24), 86% (82–91), and 182% (170–186), respectively (Table 1). Representative PET images illustrating pulmonary blood flow during the two study conditions are shown in Figure 4.

**Table 1:** Haemodynamic, ventilatory and gas exchange variables. Values are the median (IQR). Pulmonary blood flow was measured using PET, 10 min after the start of each simulation. Atelectasis was assessed by CT at approximately 20 min. All other measurements were obtained immediately after completion of the PET acquisition, approximately 18 min after simulation onset. ETCO_2_, end-tidal carbon dioxide; FiO_2_, fraction of inspired oxygen; N/A, not applicable; PAP, pulmonary arterial pressure; PET, positron emission tomography, P/F-ratio, ratio of the arterial oxygen partial pressure to the inspired oxygen fraction; SvO_2_, mixed venous oxygen saturation. ∗Hodges–Lehmann estimate of paired differences. ^†^95% confidence interval for the Hodges–Lehmann estimate. ^‡^Exact Wilcoxon signed-rank test.

	Control(normotension) (*n*=12)	Intervention(hypotension) (*n*=12)	Difference∗	95% Confidenceinterval^†^	*P*-value^‡^
**Primary outcome**					
Total atelectasis volume (ml)	51 (29–70)	76 (56–91)	20.0	9 to 38	<0.001
HR (beats min^-1^)	78 (74–104)	90 (82–99)	6	−1 to 13	0.126
MAP (mm Hg)	85 (81–93)	46 (43–49)	−40	−44 to −37	<0.001
CVP (mm Hg)	11 (9–12)	9 (7–10)	−1.5	−2.5 to −0.5	0.004
Mean PAP (mm Hg)	25 (23–27)	19 (17–21)	−6	−8 to −5	<0.001
Pulmonary blood flow					
Mean total (ml cm^−3^ min^−1^)	3.0 (2.6–3.3)	2.9 (2.5–3.5)	−0.1	−0.5 to 0.4	0.622
Mean ventral/mean total (%)	27 (22–31)	20 (18–24)	−5.1	−10 to −3	0.002
Mean interm./mean total (%)	95 (91–106)	86 (82–91)	−9.4	−14 to −4	0.018
Mean dorsal/mean total (%)	129 (104–150)	182 (170–186)	46	32 to 63	<0.001
CO (l min^−1^)	2.8 (2.5–3.4)	3.4 (3.1–3.9)	0.6	−0.1 to 1.3	0.065
Plateau pressure (cm H_2_O)	17 (16–18)	17 (14–19)	0	−0.5 to 0.5	0.938
ETCO_2_ (%)	6.0 (5.8–6.1)	5.8 (5.6–6.0)	−0.2	−0.4 to −0.1	0.003
FiO_2_	0.3	0.3	N/A	N/A	N/A
PaO_2_ (kPa)	15.9 (13.2–17.7)	8.7 (7.1–11.4)	−6.4	−7.4 to −5.3	<0.001
P/F-ratio (kPa)	53 (44–59)	29 (24–38)	−21	−25 to −18	<0.001
PaCO_2_ (kPa)	5.8 (5.6–5.9)	6.2 (6.1–6.5)	0.6	0.3 to 0.8	0.003
SvO_2_ (%)	48 (44–56)	43 (27–53)	−11	−19 to −1.3	0.023
Venous admixture (%)	5.0 (2.8–7.3)	19 (11–23)	13	8 to 18	<0.001
pH	7.44 (7.42–7.44)	7.41 (7.39–7.42)	−0.0	−0.1 to −0.0	<0.001
Lactate mM	1.4 (1.3–1.8)	1.7 (1.4–2.1)	0.2	0.0 to 0.4	0.045

**Fig 5:**
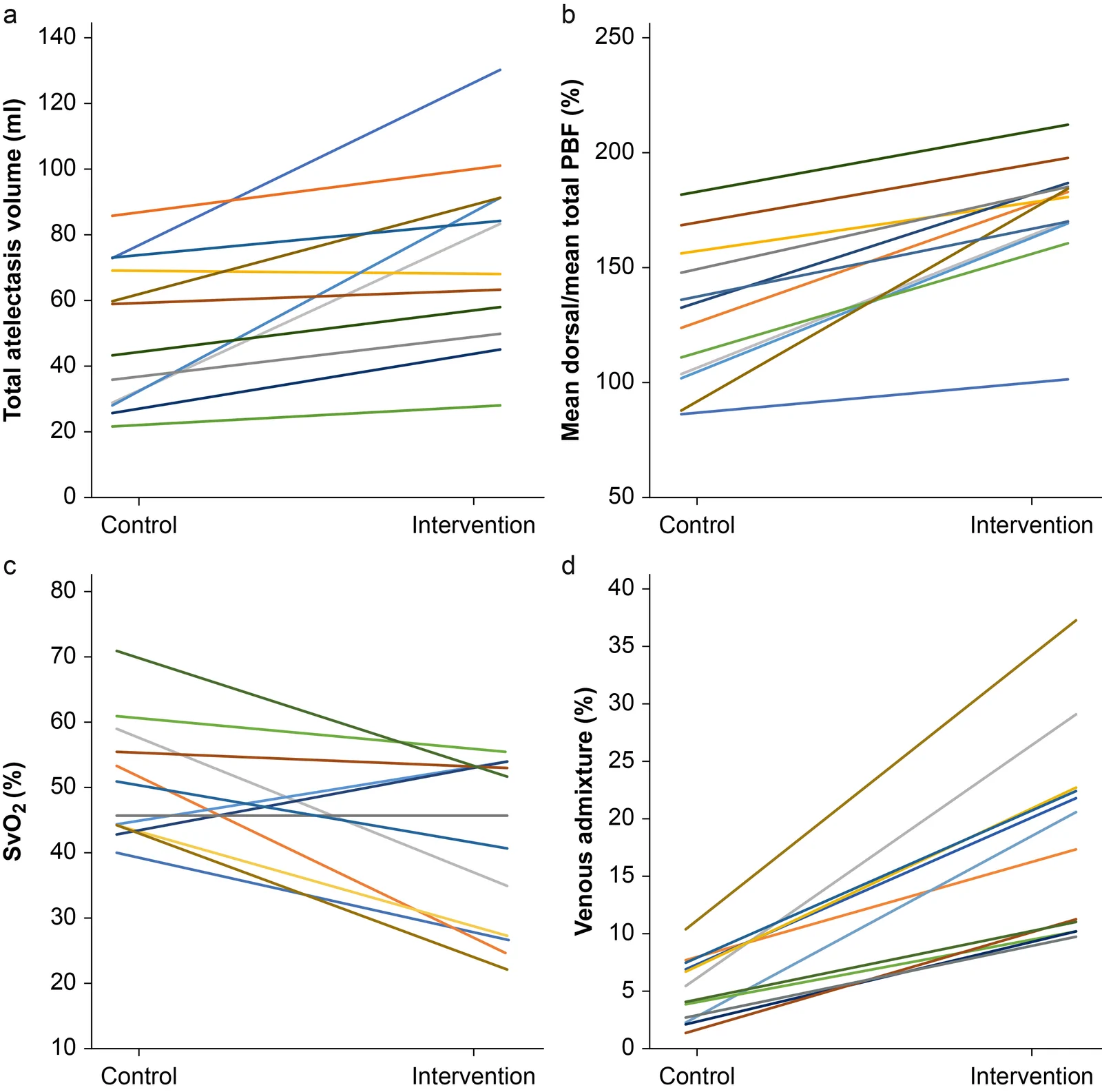
Individual (*n*=12) changes in total atelectasis volume (a), ratio of mean dorsal to mean total pulmonary blood flow (b), mixed venous oxygen saturation (SvO_2_; c), and venous admixture (d) during *control* (normotension) and *intervention* (hypotension). Each animal is represented by the same colour across panels a–d.

The hypotension condition was accompanied by a decrease in SvO_2_ and an increase in venous admixture (Table 1 and Fig. 5).

Atelectasis volume was moderately and inversely correlated with SvO_2_ (Spearman’s ρ = −0.57; 95% CI, −0.84 to −0.096) and positively correlated with venous admixture (ρ = 0.70; 95% CI, 0.49–0.85).

The individual changes in total atelectasis volume, the ratio of mean dorsal blood flow to mean total blood flow, SvO_2_, and venous admixture for each animal during control (normotension) and intervention (hypotension) are illustrated in Figure 5.

Haemodynamic, ventilatory, and gas-exchange variables are outlined in Table 1. The haemodynamic response to nitroprusside varied across subjects, and two animals did not reach the target MAP of 35 mm Hg as intended after 5 minutes of infusion. The median MAP at that time point, just before CT attenuation correction, was 36 (IQR [range] 34 to 39 [32–43]) mm Hg. The corresponding values immediately after the PET scan are presented in Table 1 and show that MAP gradually increased in most subjects despite an unchanged nitroprusside infusion rate, demonstrating physiological adaptation and/or tachyphylaxis.

Sensitivity analyses did not show signs of carryover or period effects.

## Discussion

In this porcine model, simulated induction of anaesthesia with profound nitroprusside-induced hypotension resulted in increased atelectasis formation compared with normotension. A possible explanation is redistribution of pulmonary blood flow with low SvO_2_ towards dorsal lung regions particularly prone to atelectasis.

Baseline PET imaging in supine animals demonstrated a pulmonary blood flow distribution consistent with previous observations in anaesthetised pigs^19^ and humans,^20^^,^^21^ characterised by predominant dorsal perfusion with a decreasing gradient towards ventral lung regions. Although this pattern may suggest gravity as a major determinant, other explanations have been proposed.^22^ During nitroprusside-induced hypotension, pulmonary blood flow was redistributed towards dorsal regions, further accentuating this gradient. The mechanisms underlying these changes in pulmonary circulation, including the reduction in mean pulmonary artery pressure, remain uncertain and may reflect systemic hypotension, direct pulmonary vascular effects of nitroprusside, or both.

Hypotension was also associated with a reduction in SvO_2_. Compared with humans, pigs have a right-shifted oxygen dissociation curve and therefore lower baseline SvO_2_.^23^ The further reduction during hypotension is consistent with increased tissue oxygen extraction at low arterial pressure. Lower SvO_2_ increases the potential for oxygen uptake from the alveoli into pulmonary capillaries,^7^ and delivery of less oxygen-saturated blood to dorsal lung regions prone to atelectasis may therefore promote absorption atelectasis. Low SvO_2_ may also occur in other states of reduced oxygen delivery or increased tissue extraction, such as low cardiac output and sepsis. Although both mechanisms—redistribution of pulmonary blood flow and reduced SvO_2_—are physiologically plausible, their relative contributions to the observed increase in atelectasis cannot be determined.

Over the past decade, substantial research has focused on reducing postoperative pulmonary complications through intraoperative lung-protective ventilation. Central to these strategies is preventing atelectasis, which develops shortly after induction of general anaesthesia in most patients and increases ventilation heterogeneity and injurious lung stress.^24^ Maintaining lung aeration allows ventilation with lower driving pressures and reduced mechanical strain. Effective prevention, however, likely requires a better understanding of the mechanisms underlying atelectasis formation. The present findings provide additional insight and, together with evidence from other organ systems,^25^, ^26^, ^27^ support the importance of haemodynamic stability during anaesthesia.

Strengths of this study include the consistency of the findings and their physiological coherence, further supported by results from prespecified secondary hypotheses. The crossover design, with each animal serving as its own control, enhances internal validity. Potential carryover effects were mitigated through standardised recruitment manoeuvres to re-establish baseline conditions before each intervention, and residual atelectasis was quantified and subtracted from subsequent volume measurements. However, unmeasured period-related effects cannot be fully excluded and could theoretically have influenced the magnitude of the observed differences between conditions.

Several limitations should be acknowledged. First, the use of an animal model limits generalisability to humans. Nevertheless, identifying hypotension as a contributor to atelectasis formation may have clinical relevance, particularly as direct mechanistic studies in patients would be ethically infeasible.

Second, hypotension was induced with nitroprusside, and the extent to which this reflects induction-related hypotension caused by anaesthetic agents such as propofol is uncertain, as nitroprusside may have exaggerated the physiological consequences of hypotension. For example, although atelectasis increases venous admixture through shunt,^28^ the pronounced increase observed in this study (Table 1 and Fig. 5) was likely driven less by the increase in atelectasis than by inhibition of hypoxic pulmonary vasoconstriction induced by nitroprusside.^29^

Third, the absolute atelectasis volumes were small, and the confidence intervals suggest that hypotension may have only a modest effect in this experimental setting. However, because atelectasis represents compressed lung tissue with substantially higher density than aerated lung, measured volumes underestimate the corresponding loss of aerated lung volume, which has been reported to be approximately fourfold greater.^30^ Thus, a median atelectasis volume of 76 ml may correspond to roughly 300 ml of lost aerated lung volume, equivalent to about 25% of total lung volume in pigs of similar size.^12^ The pigs studied were young and had healthy cardiovascular systems with substantial physiological reserve and adaptive capacity. In contrast, older patients, particularly those receiving antihypertensive therapy, are at greater risk of hypotension during induction of anaesthesia and may have reduced capacity to compensate for acute circulatory changes. These factors may partly explain the markedly greater atelectasis reported in patients with profound postinduction hypotension in clinical studies,^9^^,^^10^ observations that provided the rationale for the present investigation.

In summary, in a porcine model of anaesthesia induction, profound hypotension induced by nitroprusside resulted in increased atelectasis compared with normotension. These findings suggest that circulatory changes during induction contribute to atelectasis and support the importance of haemodynamic stability during anaesthesia.

## Authors’ contributions

Study conceptualisation and hypothesis generation: EÖ, LE

Study design: EÖ, LE, PW

Study methodology: EÖ, LE, MLu, MLi, GP

Study conduct: EÖ, LE

Data acquisition (CT and PET): JS

Data analysis: CW, MLu, JS, EÖ, LE

Statistical analysis: EÖ, PW

Writing - original draft: EÖ

Writing – review and editing: LE, all co-authors

## Data availability statement

Data supporting the findings of this study are available from the corresponding author upon reasonable request.

## Declaration of Generative AI and AI-assisted technologies in the writing process

During the preparation of this work the authors used ChatGPT (OpenAI) to assist with language editing of author-written text. After using this tool, the authors reviewed and edited the text as needed and take full responsibility for the content of the publication.

## Funding

The Swedish Society of Medicine (SLS-998295); Centre for Clinical Research Västmanland, Västerås, Sweden (LTV-1026688).

## Declarations of interest

MLu is co-founder, stockholder and consultant of Medtrace Pharma A/S. The other authors declare no competing interests.
